# Islet Endothelial Activation and Oxidative Stress Gene Expression Is Reduced by IL-1Ra Treatment in the Type 2 Diabetic GK Rat

**DOI:** 10.1371/journal.pone.0006963

**Published:** 2009-09-09

**Authors:** Grégory Lacraz, Marie-Hélène Giroix, Nadim Kassis, Josiane Coulaud, Anne Galinier, Christophe Noll, Mélanie Cornut, Fabien Schmidlin, Jean-Louis Paul, Nathalie Janel, Jean-Claude Irminger, Micheline Kergoat, Bernard Portha, Marc Y. Donath, Jan A. Ehses, Françoise Homo-Delarche

**Affiliations:** 1 Laboratory of Biology & Pathology of Endocrine Pancreas, Functional and Adaptive Biology Unit-CNRS EA 7059, University Paris-Diderot, Paris, France; 2 CNRS UMR 5241, P. Sabatier University, Institut L. Bugnard, Toulouse, France; 3 Laboratory of Gene Dysregulation & Differentiation, Functional and Adaptive Biology Unit-CNRS EA 7059, University Paris-Diderot, Paris, France; 4 Department of Genetic Medicine and Development, CMU, University of Geneva, Geneva, Switzerland; 5 Merck Serono, Chilly-Mazarin, France; 6 AP-HP, Hôpital Européen Georges Pompidou, Biochemistry Laboratory, Paris, France; 7 Division of Endocrinology, Diabetes & Nutrition, University Hospital of Zürich, Zürich, Switzerland; University of Bremen, Germany

## Abstract

**Background:**

Inflammation followed by fibrosis is a component of islet dysfunction in both rodent and human type 2 diabetes. Because islet inflammation may originate from endothelial cells, we assessed the expression of selected genes involved in endothelial cell activation in islets from a spontaneous model of type 2 diabetes, the Goto-Kakizaki (GK) rat. We also examined islet endotheliuml/oxidative stress (OS)/inflammation-related gene expression, islet vascularization and fibrosis after treatment with the interleukin-1 (IL-1) receptor antagonist (IL-1Ra).

**Methodology/Principal Findings:**

Gene expression was analyzed by quantitative RT-PCR on islets isolated from 10-week-old diabetic GK and control Wistar rats. Furthermore, GK rats were treated s.c twice daily with IL-1Ra (Kineret, Amgen, 100 mg/kg/day) or saline, from 4 weeks of age onwards (onset of diabetes). Four weeks later, islet gene analysis and pancreas immunochemistry were performed. Thirty-two genes were selected encoding molecules involved in endothelial cell activation, particularly fibrinolysis, vascular tone, OS, angiogenesis and also inflammation. All genes except those encoding angiotensinogen and epoxide hydrolase (that were decreased), and 12-lipoxygenase and vascular endothelial growth factor (that showed no change), were significantly up-regulated in GK islets. After IL-1Ra treatment of GK rats *in vivo*, most selected genes implied in endothelium/OS/immune cells/fibrosis were significantly down-regulated. IL-1Ra also improved islet vascularization, reduced fibrosis and ameliorated glycemia.

**Conclusions/Significance:**

GK rat islets have increased mRNA expression of markers of early islet endothelial cell activation, possibly triggered by several metabolic factors, and also some defense mechanisms. The beneficial effect of IL-1Ra on most islet endothelial/OS/immune cells/fibrosis parameters analyzed highlights a major endothelial-related role for IL-1 in GK islet alterations. Thus, metabolically-altered islet endothelium might affect the β-cell microenvironment and contribute to progressive type 2 diabetic β-cell dysfunction in GK rats. Counteracting islet endothelial cell inflammation might be one way to ameliorate/prevent β-cell dysfunction in type 2 diabetes.

## Introduction

The endothelium plays an important role in the regulation of hemostasis, blood flow, maintenance of vascular architecture and mononuclear cell migration, all of primary significance in atherogenesis. The diabetic state is well known to be associated with macrovascular complications such as atherosclerosis and medial calcifications that lead to increased risk of cardiovascular disease [1]. In addition, a diabetic-specific microvascular disease is at work in the retina (retinopathy), kidney (glomerulopathy) and vasa nervorum (neuropathy) [2].

Until now, the pancreatic islets have been only rarely considered as being a possible “end-organ” of type 2 diabetes (T2D). However, the islet has been shown to undergo significant remodelling concurrently or even earlier than other end-organs in T2D [3], [4]. Indeed, some studies have demonstrated the presence of fibrosis in various spontaneous T2D animal models, and also the presence of amyloid deposits in type 2 diabetic patients [3], [5]–[7]. Moreover, we showed recently that inflammation is present in the islets of type 2 diabetic animal models and in humans [6], [8], [9]. In the spontaneously diabetic Goto-Kakizaki (GK) rat, we suggested that these islet alterations were reminiscent of microangiopathy [6], [7]. Others, using electron microscopy, have described signs of microangiopathy in young but normoglycemic Zucker fatty rats and in the db/db mouse [10], [11].

Hyperglycemia and some other associated metabolic derangements (increased free fatty acids (FFA) and/or insulin resistance), which may precede hyperglycemia, mediate abnormal endothelial cell (EC) function via increased oxidative stress (OS), disturbances of intracellular signal transduction (such as protein kinase C activation) and activation of receptors for advanced glycation end-products (RAGE) [1]–[3], [12]–[14]. These molecular events lead to: 1) decreased nitric oxide (NO) availability associated with increased levels of endothelin-1 (ET-1) and angiotensin II (A-II) with resulting vasoconstriction and its consequences on blood flow and vascular smooth muscle cell (VSMC) growth; 2) activation of transcription factors such as nuclear factor kappa B (NF-κB) and activator protein-1 (AP-1), which increases the expression of cellular adhesion molecules such as intercellular adhesion molecule-1 (ICAM-1) and vascular cell adhesion molecule-1 (VCAM-1). It also stimulates the production of cytokines, for example, IL-1 and chemokines, such as monocyte/macrophage chemoattractant protein (MCP-1), leading to local inflammation, leukocyte attraction/migration and reactive oxygen species (ROS) production; 3) increased production of prothrombotic/dysfibrinolytic factors, such as tissue factor and plasminogen activator inhibitor-1 (PAI-1), which induce hypercoagulation and platelet aggregation [1], [15].

Overall vascular function is dependent upon the balance of pro-/antioxidant mechanisms, which determines endothelial function [14]. Considerable evidence demonstrates that OS plays a central role in the pathology of diabetes [16]–[18]. Among other factors including obesity and insulin resistance, hyperglycemia alone is able to directly induce OS [19]–[21]. Diabetes starts at around 4 weeks of age (weaning) in GK rats. After 3 months of mild hyperglycemia, GK islets show an upregulation of the gene encoding thioredoxin-interacting protein [6], which is induced by hyperglycemia and inhibits thioredoxin antioxidant function [22]. Also, ROS are involved in the mechanism of action of pro-inflammatory cytokines, such as IL-1β and tumor necrosis factor-α (TNF-α), known to be produced by EC and to target them [23]–[28]. Therefore, based on the data presented above, we hypothesized that T2D islet inflammation might have originated from EC activation.

Here, we assessed in 10-week-old diabetic GK rats and Wistar controls: 1) different circulating parameters linked to EC dysfunction, OS, inflammation, and atherosclerosis; 2) the expression of selected genes known to be involved in EC activation in freshly isolated islets; 3) the effect of 1-month treatment with IL-1Ra on islet expression of genes linked to endothelium, OS and extracellular matrix proteins, and islet vascularization and fibrosis. IL-1Ra has already been used in T2D and atherosclerosis [29]–[31]. Our data showed that endothelial activation is present in islets from 10-week-old diabetic GK rats, concomitantly with inflammation/OS. Moreover, IL-1Ra *in vivo* dampened these events, ameliorating islet vascularization, reducing fibrosis and improving glycemia.

## Results

### Dyslipidemia and signs of systemic OS in diabetic GK rats

Metabolic parameters for 10-week-old male control Wistar and diabetic GK rats are summarized in Table 1. The body weight of diabetic animals was significantly lower than controls. They displayed mildly but significantly elevated fed blood glucose and insulin levels [32]. They also showed hyperleptinemia and significantly increased circulating levels of triglycerides, FFA, total cholesterol and high density lipoproteins (HDL) cholesterol. Their total cholesterol/HDL cholesterol ratio was similar to that of Wistar rats. The glutathione redox state was significantly lower in GK than Wistar red blood cells (RBC), with comparable equivalent reduced glutathione contents (Eq GSH). Plasma α-tocopherol level was significantly higher in GK animals than controls. Concomitantly, the plasma homocysteine level, an independent risk factor in the development of atherosclerosis [33], [34], was significantly lower. Moreover, the activity of paraoxonase-1 (PON-1), an HDL-associated lipo-lactamase, whose activity is negatively correlated with homocysteine [35], was signicantly higher in diabetic than control animals. However, circulating cytokines/chemokines levels, such as GRO1/KC (or CXCL1, the rodent equivalent of IL-8), MCP-1 (CCL2), MIP-1α (macrophage inflammatory protein-1α or CCL3) and IL-6 were not significantly different at this age between both groups. Therefore, in addition to mild basal hyperglycemia, 10-week-old adult GK rats also exhibited hyperlipidemia, blood OS (as reflected by oxidized RBC glutathione redox state), but had already mounted blood antioxidant defense (high α-tocopherol level and PON-1 activity).

**Table 1:** Metabolic data for 10-week-old control Wistar and diabetic GK male rats.

Parameters	Wistar	GK
Body weight (g)	384±10	277±8^*^
Glucose (mM)	5.9±0.3	8.3±0.4^*^
Insulin (pM)	184±43	440±115^*^
Leptin (pM)	250±33	360±17^*^
Triglycerides (mM)	1.5±0.1	2.1±0.1^*^
FFA (mM)	0.5±0.0	0.7±0.0^*^
Total cholesterol (mM)	1.6±0.1	2.0±0.0^*^
HDL cholesterol (mM)	1.1±0.1	1.4±0.0^*^
Cholesterol/HDL ratio	1.4±0.0	1.4±0.0
RBC glutathione redox state	93.5±1.0	80.1±3.1^*^
RBC Eq GSH content (mM)	3.9±0.3	3.8±0.3
α-Tocopherol (µM)	13.5±0.6	22.0±1.1^*^
Homocysteine (µM)	9.9±0.6	6.0±0.3^*^
PON-1 (%)	100±4	116±3^*^
GRO1/KC (pg/ml)	312±70	337±37
MCP-1 (pg/ml)	153±16	219±45
MIP-1α (pg/ml)	6.4±0.8	7.6±2.5
IL-6 (pg/ml)	79±21	224±113

Glucose, insulin, leptin, lipids, cytokine and chemokine levels were determined in serum. Alpha-tocopherol and homocysteine levels, and paraoxonase-1 (PON-1) activity were determined in plasma. Glutathione redox state (% of reduced glutathione (GSH)) and GSH content (Eq GSH) were determined in red blood cells (RBC). Glucose, insulin, leptin: n = 7 per group; lipids: n = 9 per group; cytokines/chemokines: n = 7 per group; α-tocopherol, glutathione redox state and GSH content (n = 7–13 per group) and homocysteine and PON-1: n = 7–8 per group. All parameters were assayed under fed conditions. FFA (free fatty acids); HDL: high density lipoproteins; GRO1/KC/CXCL1: rodent equivalent of IL-8; MCP-1/CCL2: monocyte/macrophage chemoattractant protein; MIP-1α/CCL3: macrophage inflammatory protein-1α; IL-6, interleukin-6. ^*^p<0.05 versus age-matched Wistar group, as analyzed by Student's *t*-test.

### Islet endothelial activation in diabetic GK rats

Hyperglycemia is well recognized to be associated with increased arterial wall inflammation, reflected by increased expression of anti-fibrinolytic components, vascular cell adhesion molecules, renin-angiotensin system (RAS) factors, agents involved in OS, vascular tone and angiogenesis, and also of cytokines, chemokines, Toll-like receptors (TLRs) and molecules involved in their signalling [1], [15], [25], [28], [36]–[38]. Therefore, we selected 32 genes encoding molecules belonging to these different classes. Expression of these genes is shown in Table 2. Twenty-eight of 32 of the selected genes were significantly over-expressed. These genes encoded the following molecules: 1) anti-fibrinolysis system: PAI-1; 2) vascular adhesion molecules: E-selectin (E-SELE), ICAM-1, platelet-endothelial cell-adhesion molecule-1 (PECAM-1) and VECAM-1; 3) RAS: angiotensin-converting enzyme-1 (ACE-1) and angiotensin receptor-1α (AGTR-1α); 4) vascular tone/OS/angiogenesis components: cyclo-oxygenase-2 (COX-2), endothelial nitric oxide synthase (eNOS), endothelin-1 (ET-1), heme-oxygenase-1 (HO-1), hypoxia-induced factor-1α (HIF-1α), NADPH-oxidase-2 (NOX-2), prostacyclin synthase; 5) cytokines and growth factors: IL-1β, IL-1Ra, IL-6, transforming growth factor-β (TGF-β), tumor necrosis factor-α (TNF-α); 6) chemokines: GRO1/KC, MCP-1, and MIP-1α; 7) cellular pathways for cytokines and TLRs: caspase-1, TLR-2, TLR-4, myeloid differentiation primary response protein MyD88, NF-κB and inductible nitric oxide synthase (iNOS). The expression of 2 genes of 32 was found to be significantly decreased, one encoding a molecule belonging to RAS: angiotensinogen and the other encoding soluble (cytoplasmic) epoxide hydrolase (sEH), involved in vascular tone, OS, inflammation and angiogenesis. Finally, the gene expression for the pro-oxidant 12-lipoxygenase (12-LOX) and the pro-angiogenic factor vascular endothelial growth factor (VEGF) was not modified. Thus, islets from 10-week-old diabetic GK rats are characterized by increased mRNA levels of molecules involved in dysfibrinolysis, endothelium cell adhesion, vascular tone, OS and inflammation.

**Table 2:** Expression of genes encoding factors involved in endothelium activation and inflammation in GK islets.

Protein names	Acronyms	mRNA levels		
		Wistar (W)	GK	Fold of W
***Dysfibrinolysis***				
Plasminogen activator inhibitor-1	PAI-1	0.4±0.1	31.5±3.2	↑ x70^*^
***Cellular adhesion molecules***				
E-selectin (CD62)	E-SELE	0.8±0.1	11.8±2.3	↑ x14^*^
Intercellular adhesion molecule-1 (CD54)	ICAM-1	0.8±0.2	5.0±0.7	↑ x6^*^
Platelet endothelial cell adhesion molecule-1 (CD31)	PECAM-1	2.5±0.5	4.0±0.3	↑ x2^*^
Vascular cell adhesion molecule-1 (CD106)	VCAM-1	0.1±0.0	9.3±1.1	↑ x62^*^
***Vascular tone/oxidative stress/angiogenesis***				
Angiotensin-converting enzyme-1	ACE-1	3.4±0.9	7.3±0.4	↑ x2^*^
Angiotensin receptor-1α	AGTR-1α	1.4±0.2	2.9±0.3	↑ x2^*^
Angiotensinogen	AGT	3.5±0.7	1.6±0.2	↓ x0.5^*^
Cyclo-oxygenase-2	COX-2	0.9±0.2	17.3±4.1	↑ x19^*^
Endothelial nitric oxide synthase	eNOS	0.9±0.1	2.4±0.1	↑ x3^*^
Endothelin-1	ET-1	0.5±0.2	1.4±0.1	↑ x3^*^
Epoxide hydrolase 2, soluble	sEH	1±0.0	0.1±0.0	↓ x0.1^*^
Heme-oxygenase-1	HO-1	1.8±0.2	45.5±0.1	↑ x25^*^
Hypoxia-induced factor-1α	HIF-1α	2.6±0.1	4.5±0.6	↑ x2^*^
12-Lipoxygenase	12-LOX	0.3±0.1	0.3±0.1	→ x1
NADPH-oxidase-2	NOX-2	0.9±0.1	2.8±0.1	↑ x3^*^
Prostacyclin synthase	PGIS	1.7±0.2	8.8±0.7	↑ x5^*^
Vascular endothelial growth factor A	VEGFA	2.1±0.2	2.3±0.1	→ x1
***Cytokines/growth factors***				
Caspase-1 or IL-1-converting enzyme	Caspase-1	1.0±0.0	2.3±0.0	↑ x2^*^
Interleukin-1β	IL-1β	0.3±0.1	4.5±0.9	↑ x15^*^
Interleukin-1 receptor antagonist	IL-1Ra	0.1±0.0	3.7±0.2	↑ x34^*^
Interleukin-6	IL-6	1.3±0.4	48.9±8.1	↑ x38^*^
Transforming growth factor-β	TGF-β	1.1±0.1	7.2±0.5	↑ x7^*^
Tumor necrosis factor-α	TNF-α	0.5±0.3	16.4±4.1	↑ x33^*^
***Chemokines***				
Chemokine GRO/KC (rodent analog of IL-8)	KC or CXCL1	0.6±0.2	73.1±15.5	↑ x113^*^
Monocyte chemoattractant protein-1	MCP-1 or CCL2	0.3±0.0	66.3±11.9	↑ x204^*^
Macrophage inflammatory protein-1α	MIP-1α or CCL3	1.0±0.3	44.7±5.6	↑ x46^*^
***Toll-like receptor/intracellular pathways***				
Inductible nitric oxide synthase	iNOS	3.2±0.5	21.9±1.9	↑ x7^*^
Myeloid differentiation primary response protein MyD88	MyD88	0.8±0.2	1.7±0.1	↑ x2^*^
Nuclear factor kappa B (p65)	NF-κB	1.0±0.1	2.4±0.1	↑ x2^*^
Toll-like receptor-2	TLR-2	1.0±0.1	4.4±1.1	↑ x4^*^
Toll-like receptor-4	TLR-4	0.4±0.1	1.9±0.2	↑ x4.8^*^

Total RNA was extracted from freshly isolated islets of 2.5-month-old male Wistar and GK rats and quantitative RT-PCR was performed for the indicated genes and normalized to a housekeeping gene (rpL19 or Ef1a). Data are means±SEM of 5–6 different islet isolations per group except for caspase-1 and epoxide hydrolase-2 (n = 3).^*^p<0.05 using Student's *t*-test.

### Beneficial effect of IL-1Ra on glycemia and expression of genes selected for endothelial activation, OS and ECM, and on vascularization and fibrosis in GK islets

Endothelial activation, OS and mechanisms of cytokine action are tightly linked [23]–[28]. IL-1β, produced by EC among other cells, is involved in microangiopathy/atherogenesis [28], [39], [40]. Therefore, we administered IL-1Ra, its natural antagonist, s.c. twice daily at 50 mg/kg (i.e. 100 mg/kg/day) to GK rats, from 4 weeks (weaning and onset of diabetes) to 8 weeks of age. At the end of treatment, IL-1Ra-treated GK rats showed a significantly lower glycemia than GK saline (controls): 8.8±0.3 mM (n = 7) and 7.9±0.1 mM (n = 8, p<0.05), respectively.

**Figure 1:**
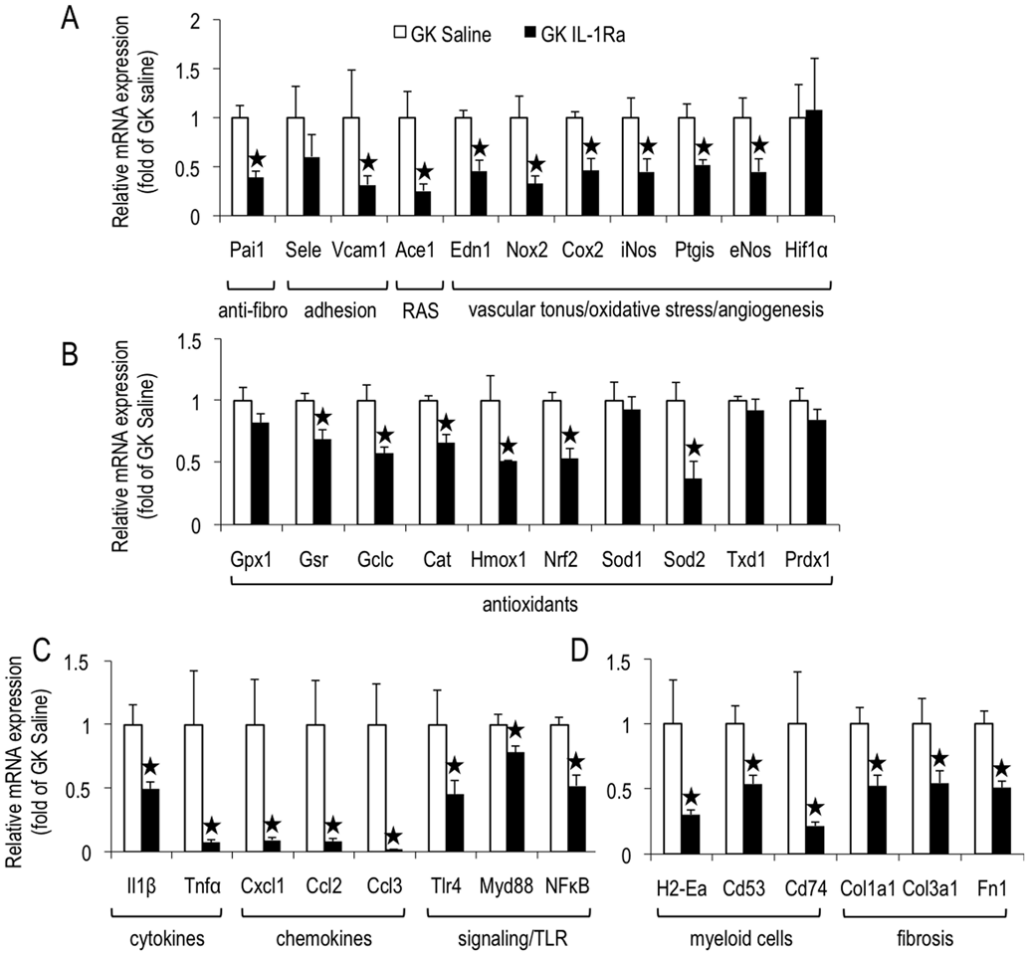
IL-1Ra treatment reduces the expression of most of the selected genes for endothelial activation, oxidative stress, myeloid cells, and fibrosis in GK islets. Pancreatic islets were isolated from GK rats following 1-month-treatment with IL-1Ra by s.c. injections (GK saline n = 6, GK IL-1Ra (100 mg/kg/day), n = 5). For each animal, total RNA was extracted from isolated islets and quantitative RT-PCR was performed for the indicated genes, and expressed relative to GK saline. ^*^p<0.05 using Student's *t*-test.

GK rat IL-1Ra treatment down-regulated 80% of selected genes. As shown in Fig. 1A, IL-1Ra treatment significantly (except otherwise stated) decreased GK islet expression of genes encoding: PAI-1 (−61%), VCAM-1 (−68%, ns), ACE-1 (−74%), ET-1 (Edn1, −54%), NOX-2 (−67%), COX-2 (−60%), iNOS (−54%), prostacyclin synthase (Ptgis, −48%), and eNOS (−55%) *vs* Wistar controls. However, IL-1Ra treatment did not significantly reduce islet endothelial gene expression for E-selectin (Sele) and HIF-1α.

The effects of IL-1Ra treatment on genes encoding antioxidant molecules are shown in Fig. 1B. Beta-cells appear to be especially vulnerable to ROS attacks due to their low levels of antioxidant enzymes [41], [42]. To compensate for such vulnerability, diabetic β cells may upregulate antioxidant genes *in vivo* [43], as we recently showed in GK islets [44]. Here, IL-1Ra treatment of GK rats was able to down-regulate the expression of some of these genes but not all of them. First, the gene expression of one of the main stress-activated mitochondrial enzymes, superoxide dismutase 2 was decreased (Sod2, −63%). This enzyme represents the first-line of defense against superoxide anions generated by the mitochondria. However, IL-1Ra did not modify the expression of Sod1 gene. IL-1Ra further reduced significantly catalase (Cat, −34%), but not glutathione peroxidase 1 (Gpx1), nor thioredoxin 1 (Txd1), or peroxiredoxin 1 (Prdx1). Those genes encode molecules involved in further reduction of superoxide-derived compounds (H_2_O_2_). IL-1Ra treatment down-regulated the expression of genes encoding γ-glutamylcysteine ligase catalytic subunit (Gclc, −42%) and glutathione reductase (Gsr, −31%), both of which contribute to maintain the content of GSH, an antioxidant thiol, whose mechanisms include: 1) an antioxidant potential mediated by the peroxidase-coupled reaction; 2) regulation of cellular sulfhydryl status and redox equilibrium; 3) regulation of expression/activation of redox-sensitive transcription factors induced by stress-evoked responses [45]. IL-1Ra treatment also significantly lowered the gene encoding HO-1 (Hmox1, −49%), an antioxidant induced by supra-physiological glucose concentrations [46], inflammation [47], cytokines [48], [49], and oxidative low density lipoproteins (LDL) [50]. Finally, IL-1Ra treatment down-regulated the expression of NF-E2-related factor (Nrf2, −47%), which drives the expression of several genes, such as Gclc and Hmox1 [51].

As shown in Fig.1C, IL-1Ra reduced mRNA islet levels of TLR4 (−55%), MyD88 (−22%) and NF-κB (−48%), in addition to mRNA down-regulation of various cytokines/chemokines (including IL-1β), as shown elsewhere [52]. Not surprisingly therefore, IL-1Ra treatment also down-regulated the expression of genes encoded by macrophages or immature myeloid cells, which infiltrate GK islets at this age [6]. For example, the mRNA levels of MHC (major histocompatibility complex) class II (H2-Ea), CD53, and CD74 (macrophage inhibitory receptor or MIF) were strongly reduced: −70%, −40%, and −78%, respectively). Moreover, the genes encoding the main three extracellular matrix proteins constituting GK islet fibrosis (collagen I, collagen III and fibronectin, whose genes were over-expressed in GK *vs* Wistar islets [6]) were down-regulated after IL-1Ra treatment (Col1a1, −48%, Col3a1, −45%; Fn1, −49%, respectively) (Fig. 1D).

**Figure 2:**
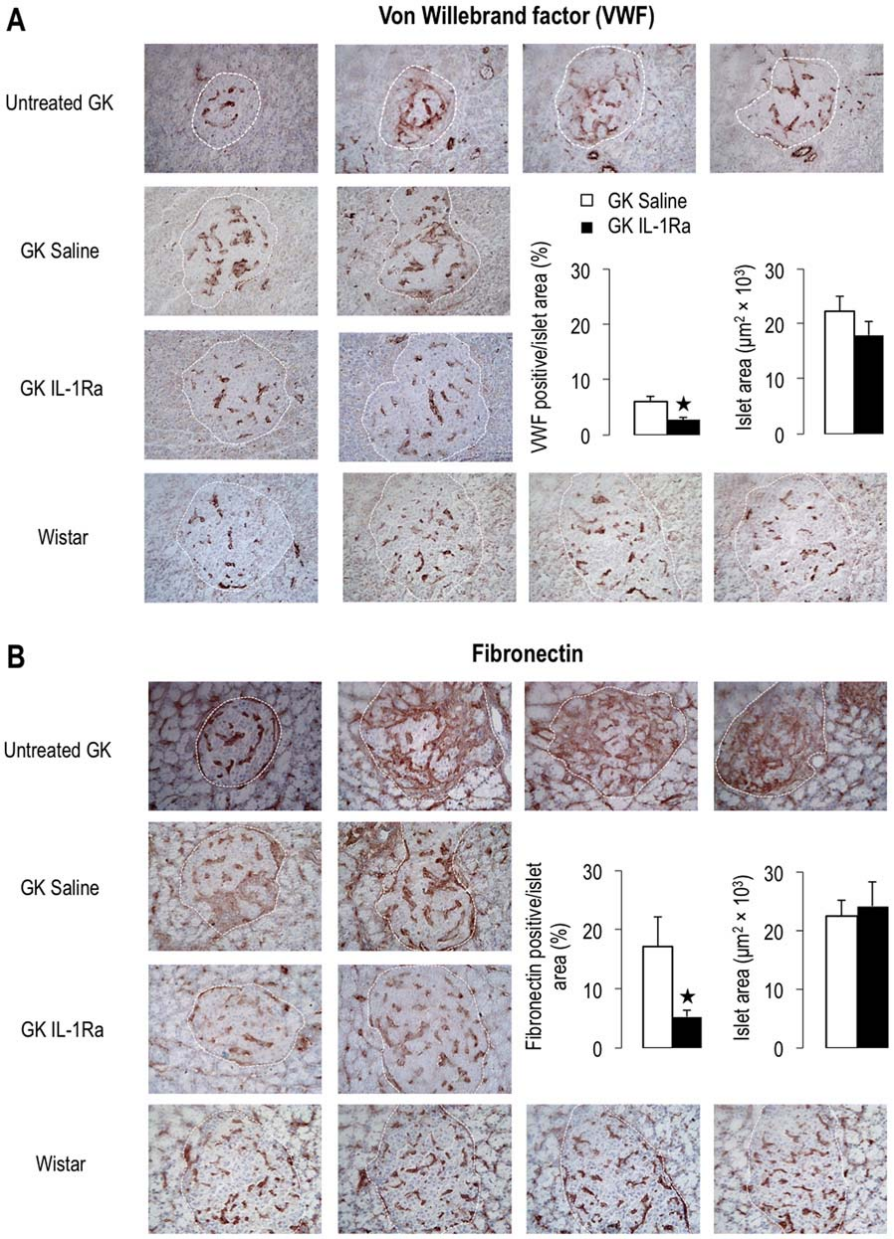
IL-1Ra treatment improves vascularization and reduces fibrosis in GK islets. Immunohistochemistry was performed for von Willebrand factor (VWF) (A) and fibronectin (B) in pancreas of young adult untreated Wistar and GK rats, and of s.c. saline- or IL-1Ra-treated-GK rats. The border of each islet is defined by the dashed line. As shown in panel A, VWF-labeled islets from untreated GK rats are extremely heterogeneous in terms of vascularization and extent of fibrosis, when compared to Wistar controls. In saline- and IL-1Ra-treated GK rats, immunolabeled islet area for VWF or fibronectin was quantified for each islet and expressed as to the corresponding islet surface (n = 3 GK rats for both treatment groups, n = 25–40 islets). Islets analyzed for quantification showed unchanged islet area between treatment groups. ^*^p<0.05 using Student's *t*-test.

Finally, we performed immunohistochemistry for von Willebrand factor (VWF), an EC marker, and for fibronectin, a main component of GK islet fibrosis, also produced by EC [6]. Von Willebrand factor and fibronectin islet labeling examples are shown in figure 2 (panels A and B). As previously described [6], islets of adult GK rats are extremely heterogeneous, compared to age-matched Wistar islets: they showed different degrees of endothelial alteration and fibrosis. More precisely, GK islet vascularization appears more or less hypertrophied or even greatly disorganized. One month of IL-1Ra treatment significantly reduced labeling of GK islet alterations, as shown for both VWF and fibronectin (−53% and −69%, respectively).

## Discussion

The origin of the recently recognized islet inflammation in T2D is still an open question. A few studies in various spontaneous T2D animal models (Zucker diabetic fatty, Otsuka Long-Evans Tokushima fatty and Torii rats, and db/db mice) indicated islet vessel alterations (leakage, hemorrhage), anomalies of blood flow, microangiopathy [10], [11], [53], [54] or even amyloid deposits along vessels in type 2 diabetic patients [5]. We hypothesized that islet inflammation might have originated from EC activation (for review, see [7]). Using a molecular approach, we demonstrate here, for the first time, that 10-week-old diabetic GK rats show increased markers of endothelial microvessel activation associated with inflammation in islets. Moreover, *in vivo* IL-Ra treatment reduced most of these molecular and vascular alterations, islet fibrosis and glycemia.

Endothelial dysfunction has already been described in old GK rat macrovessels (mesenteric artery, thoracic aorta and cerebral arteries) [55]–[59]. These studies showed adhesion molecule gene overexpression, pronounced renal perivascular monocyte/macrophage infiltration, increased vascular OS, and RAS and ET-1 involvement. Moreover, GK macrophages exhibit a pro-inflammatory phenotype associated with the pathogenesis of atherogenesis [60]. Alpha-lipoic acid, AGTR-1 and ET-1 antagonists have been shown to provide vasoprotective effects in these macrovessels [55], [57]–[59]. Here, we extend the observation to alterations of the islet microvascular bed in an early stage of the GK disease development.

As expected, 8–10-week-old diabetic GK rats showed mild hyperglycemia, hyperinsulemia and hyperlipidemia. However, it should be mentioned that, at this age (around onset of insulin resistance), hyperinsulinemia may be observed or not from one batch of GK rats to another. Our data confirm that male diabetic GK rats exhibit increased levels of triglycerides, FFA, cholesterol and HDL [61], [62]. Notably, extensive physiological screening in both sexes of congenics revealed the existence of GK variants at the locus Nidd/gk5, independently responsible for significantly enhanced insulin secretion and altered cholesterol metabolism [61]. GK rats had marked hyperleptinemia, classically associated with obesity and/or hyperinsulinemia [63]. Concomitantly, peripheral OS, as reflected by oxidized RBC glutathione redox state, was present in GK rats. Patients with high circulating homocysteine levels, an independent risk factor of atherosclerosis development, have an impaired ability to induce cholesterol efflux from macrophages [33], [34], [64]. Homocysteine, unexpectedly lower in diabetic rats than in controls, might be responsible for the higher systemic GK cholesterol levels. PON-1 is an antioxidant agent and anti-atherogenic HDL-associated enzyme, which prevents LDL and HDL oxidation [65], [66]. Homocysteine has been demonstrated to be negatively correlated to the plasma activity of PON-1 in a mouse model of homocysteine disorder [35]. This is also the case in diabetic GK rats, which had higher plasma PON-1 arylesterase activity than Wistar. In this regard, it should be noted that increasing PON-1 in mice attenuated diabetes-induced macrophage OS, diabetes development and decreased mortality [67]. Therefore, high circulating PON-1 activity together with enhanced α-tocopherol (vitamin E) in 10-week-old diabetic GK rats, might suggest that these rats had already installed a systemic antioxidant defense in the lipophilic plasma compartment. The latter might be characterized by some degree of leptin resistance, because of the decreasing effect of leptin on plasma PON-1 activity in Wistar rats [68], and hyperleptinemia in GK rats. This might explain why, despite high circulating levels of the pro-inflammatory/pro-oxidant leptin, systemic cytokine/chemokine levels were similar in GK rats and Wistar controls, while the levels of some of them was high in GK rats before weaning (see below).

**Figure 3:**
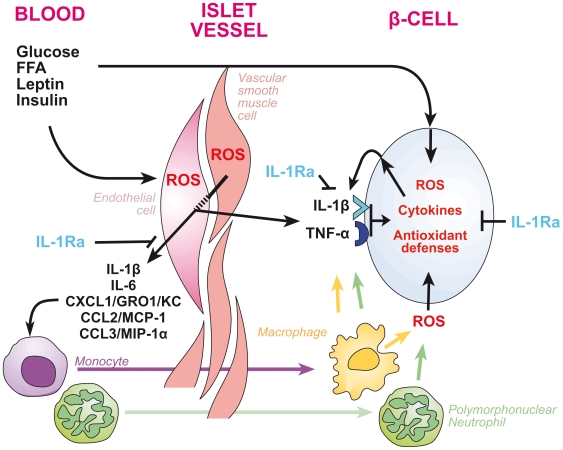
Proposed model illustrating the islet endothelial dysfunction and oxidative stress surrounding β cells in GK rats. Elevated glucose, FFA and possibly cytokines induce endothelial activation at the islet level by eliciting reactive oxygen species (ROS) production and cytokine/chemokine release by endothelial cells and vascular smooth muscle cells. Once released, chemokines (CXCL1, CCL2, CCL3) attract/retain immune cells (monocytes, neutrophils), which further induce ROS and cytokine production around β cells. In addition, cytokines as well as metabolic factors (high glucose and FFA) may act directly on β cells to increase intracellular cytokines and ROS production with consecutive antioxidant defense response. Antagonizing IL-1 by IL-1Ra may inhibit endothelial activation/dysfunction and subsequent immune cells attraction/activation. IL-1Ra may also blunt IL-1 signaling in β cells and subsequent ROS production and antioxidant defense response. CXCL1 (GRO1/KC, rodent equivalent of IL-8), CCL2 (MCP-1); CCL3 (MIP-1α); FFA (free fatty acids); IL-1β, interleukin-1β; IL-1Ra, interleukin-1 receptor antagonist; IL-6, interleukin-6; TNF-α, tumor necrosis factor-α.

Molecular signs of GK islet endothelial activation and OS might not only derive from mild chronic hyperglycemia, but also from associated metabolic disorders, such as increased circulating FFA and cholesterol levels and/or insulin resistance, which can precede hyperglycemia [1]–[3], [12]–[14]. Hyperglycemia alone is recognized for a long time to be deleterious for EC [1], [2], [17], while it induces pro-inflammatory cytokine/chemokine expression in monocytes and increases their adhesion to EC [69], where hyperglycemia-induced ROS toxicity is dependent on paracrine factor release, like cytokines [70]. High levels of FFA signaling through TLR receptors, cholesterol, leptin and A-II are also able to stimulate cytokine/chemokine release from EC and/or vascular smooth muscle cells, consequently increasing vascular OS [63], [71], [72] (Fig. 3). Then, inflammatory cells migrate and produce cytokines/chemokines and ROS [73], which might alter β-cells. Also, β-cells are able to produce IL-1 and IL-Ra in the presence of glucose, FFA and/or leptin [74]. A vicious cycle is therefore initiated that will alter islet blood flow and β-cell function [7], unless the islets are able to mount defense mechanisms.

Notably, islet capillary alterations and hemorrhage and signs of microangiopathy are already present in normoglycemic Torii and Zucker fatty rats, before onset of diabetes [11], [53]. In Torii rats, hyperlipidemia precedes hyperglycemia [75]. Similarly, we observed in prediabetic 7-day-old GK *vs* Wistar neonates, increased serum FFA levels and high cholesterol/HDL ratio together with elevated levels of chemokines (CXCL-1, CCL2 and CCL3) [76]. Because we also found higher circulating total cholesterol/HDL cholesterol ratio in E21.5 GK fetuses, our present hypothesis is that dyslipidemia, together with fetal GK hyperglycemia [77], initiates an islet microangiopathy/atherosclerosis process via *in utero* programming [78]. Such developmental metabolically-induced alterations may explain the high islet ROS levels associated with altered glutathione and thioredoxin-related gene expression recently observed in prediabetic 7-day-old GK neonates [44]. Thus, using oligo GEArrays targeted at endothelium and cardiovascular disease biomarkers, we showed a significant upregulation of genes encoding various inflammatory molecules [76], while the expression of genes encoding eNOS, E-selectin, VCAM-1, IL-1β, IL-1Ra, IL-6, TNF-α, TLR-4 and MyD88 (upregulated in 10-week-old GK rats) was similar in 1-week-old prediabetic GK and Wistar islets (data not shown).

After diabetes onset, however, we showed in GK islets that most of the selected 32 genes, which were up-regulated, encoded deleterious molecules involved in microangiopathy/atherosclerosis. Some molecules are more or less specific to endothelium (such as PAI-1, VCAM-1, E-selectin, PECAM-1 and eNOS), while part of endothelial activation includes inflammatory products not specific to endothelium. For example, cytokines and chemokines can be produced by different cell types, including EC, VSMC, macrophages, granulocytes, even β-cells or other endocrine cells [7]. As hypothesized, there was an over-expression of genes encoding: anti-fibrinolytic agent, cellular adhesion molecules, cytokines/chemokines, growth factors and caspase 1, which cleaves pro-IL-1 and pro-IL-18 in their active forms [79]; and finally TLRs, which are stimulated by FFA and involved in atherosclerosis [37], and molecules involved in TLR and IL-1 signaling: MyD88, NF-κB and iNOS. The majority of molecules selected here are known to be modulated at the transcriptional level. However, a few of them, particularly NF-κB and HIF-1α, are regulated at the post-transcriptional and even post-translational level. Therefore, their mRNA levels do not correlate systematically with their transcriptional activity. Here, however, increased gene NF-κB expression was accompanied with more translational activity, because of its association with gene upregulation of IL-1 and iNOS, 2 molecules known to be under NF-κB regulation. In addition, mRNA levels of other genes under NF-κB regulation (antioxidant/antiapoptotic genes) were also elevated in 10-week-old GK islets [80].

The GK islet iNos overexpression should be underlined on the basis of the following data: 1) studies using iNOS-deficient mice showed that iNOS plays an important role in the pathogenesis of vascular lesions characteristic of the early stages of diabetic retinopathy, preventing leukostasis [81]; 2) granulocytes infiltrate diabetic GK islets [6], express iNOS and produce ROS [73]; 3) granulocytes induce cardiomyocyte injury after myocardial ischemia/reperfusion by an iNOS-derived OS and peroxynitrite-mediated mechanism [82]; 4) in the aortic tissue of diabetic GK rats, superoxide production is increased but NO bioavailability decreased [56]. This is associated with elevated eNOS protein expression and low levels of its cofactor tetrahydrobiopterin, increased nitrosylated protein content and expression of the superoxide-generating enzyme NADPH oxidase (particularly, NOX-2). These data suggest that diabetes triggers ROS production from the NADPH oxidase, leading to tetrahydrobiopterin oxidation, eNOS uncoupling, and oxidative NO inactivation with subsequent peroxinitrite formation [83]. Moreover, increased expression of NOX-2 and eNOS mRNA and protein levels have been observed in the mesenteric arteries of streptozotocin-induced diabetic apolipoprotein-E-deficient mouse [84]. Similarly, genes encoding NOX-2 and eNOS were also up-regulated in our diabetic GK islets, together with elevated eNOS protein level (unpublished data) and increased GK islet NO production [85], therefore strenghtening eNOS uncoupling. Finally, it should be noted that: 1) eNOS expression is stimulated by hypercholesterolemia, TNF-α, TGF-β and hypoxia; 2) NADPH oxidase expression is stimulated by glucose, FFA and cytokines in rat pancreatic islets and a clonal β-cell line [86]; and 3) high glucose levels downregulate the number of monocytic calveolae, which mediate the intracellular lipid transport, through NADPH oxidase-induced OS [87]. The gene encoding the pro-inflammatory/pro-oxidant COX-2 enzyme was strongly up-regulated in GK islets. Notably, inhibition of COX-2 gene or its deletion in macrophages protects against atherosclerosis [88]. Regarding β-cell function, COX-2 over-expression could be deleterious, because its selective inhibition is able to enhance glucose-induced insulin secretion through a reduction of prostaglandin E2 [89].

Among other genes that were found to be up-regulated in diabetic GK islets are those encoding several vasoconstrictor agents, such as ET-1 and RAS-linked molecules. Endothelins activate NADPH oxidases, thereby increasing superoxide production and OS, and consequently leading to endothelial dysfunction [90], [91]. ET-1 has also been shown to stimulate *in vitro* the release of IL-1β, TNF-α and IL-6 from monocytes [92] and has marked vasoconstrictor effect on mouse pancreatic islet vasculature, either *in vivo* or in vascularly perfused islets [93]. As already mentioned, circulating ET-1 levels are elevated in aged GK rats and ET-1 antagonists ameliorate their macrovessel alterations [58]. The presence of various RAS components has been described at the islet level [94], and A-II-mediated signal through AGTR-1 involves NADPH activation, superoxide production and eNOS uncoupling [72]. In the diabetic retina, A-II induces leukostasis *via* NADPH activation [95]. AGT, ACE, and or AGTR-1 mRNA and protein levels are elevated in arterial cells of type 2 diabetic patients, and RAS inhibition reduces the onset of T2D and prevented atherosclerosis [96]. Hypercholesterolemia stimulates angiotensin peptide synthesis and contributes to atherosclerosis through the AGTR-1 [97]. Moreover, in streptozotocin-induced diabetic mouse aortas, the AGTR-1 blocker candesartan or the ACE inhibitor captopril markedly attenuates eNOS-derived ROS production, while augmenting NO bioavailability, implicating eNOS recoupling [98]. In db/db mouse islets, candesartan, ameliorates β-cell function, decreases OS markers and fibrosis, and prevents EC loss [99]. As expected, GK islets showed an up-regulation of ACE, AGTR-1 genes but an AGT gene down-regulation, which could be linked to the local insulin inhibitory effect on AGT mRNA expression in EC [100].

In addition to the up-regulated genes with deleterious effects, we noted a few genes, whose modulation would be supposed to exert protective effects. This is the case for the gene encoding prostacyclin synthase (Ptgis), which is stimulated by IL-1β, TNF-α and TGF-β, and produces prostacyclin (PGI2), a potent vasodilatator agent [101]. Two other over-expressed genes, also stimulated by cytokines and hypoxia, encode molecules with antioxidant and/or pro-angiogenic effects: HO-1 and HIF-1α. HO-1, whose gene expression was markedly up-regulated in GK islets, is a potent antioxidant agent, which is able to decrease MCP-1 but increase VEGF in EC [102], inhibit their adhesion molecules expression [103] and protect them from glucose-induced apoptosis [104]. HO-1 induction improves pancreas graft survival by preventing pancreatitis after transplantation, and protects pancreatic microcirculatory dysfunction after ischemia/reperfusion in rats [105], [106]. HIF-1α, like NF-κB, is regulated at post-transcriptional and/or post-translational levels. The HIF-1α overexpression in GK islets is possibly linked to vessel alterations. We also showed that a crucial target gene of HIF-1α, Ldha (encoding lactate deshydrogenase A) was also overexpressed in GK islets [80]. HIF-1α regulates several pro-angiogenic genes, eNOS, HO-1, MIF, and VEGF [107]. Because of concomitant gene upregulation of eNOS and HO-1, it is highly probable that HIF-1α activity was also stimulated in GK islets. However, VEGF gene expression showed no change. This observation might be of importance in the context of T2D and atherosclerosis, where defects in endothelial precursor cells were recently recognized [108], [109]. GK rats might present a deficient angiogenesis due to lack of VEGF response and also possible eNOS uncoupling, because VEGF-induced-eNOS is an efficient pathway of angiogenesis [110], [111] as well as COX-2 [112]. In this context, the lack for GK rats to increase blood flow and islet mass after 60% pancreatectomy as opposed to Wistar rats should be noted [113].

IL-1Ra, the natural IL-1 antagonist, is another molecule of interest in this study for several reasons. It has been shown to: 1) play a crucial role in the prevention of inflammatory diseases; 2) counteract deleterious effects of IL-1 members involved in insulin resistance and diabetes; 3) reduce hyperglycemia and improve β-cell function in type 2 diabetic patients [29], [114], [115]. In addition, some haplotypes of the IL-1Ra gene have been found to be correlated with increased cardiovascular disease risk in patients with or without diabetes and IL-1Ra is being to be used in atherosclerosis [31], [116], [117]. Compared to Wistar, untreated GK islets showed a marked IL-1Ra gene up-regulation, which was unable to counteract spontaneously *in situ* the consequences of the concomitant strong IL-1β gene up-regulation. In this regard, on can note that: 1) a very high IL-1Ra/IL-1 ratio is necessary to counteract IL-1 effects; 2) *in situ* a very low IL-1β concentration can exert its pleiotropic action and part of the latter may become IL-1-independent with time; 3) and/or the GK islet inflammatory process could be triggered concomitantly by other factors, such as TLR activation via dyslipidemia [71]. By contrast, 100 mg/kg/day IL-1Ra treatment of GK rats was able to significantly down-regulate most selected genes for endothelial activation, all cytokines/chemokines and their pathways, myeloid cell infiltration, and ECM proteins. Concomitantly, most antioxidant gene expression was down-regulated, particularly those known to be activated by IL-1 at the EC level [118] and serum level of the antioxidant PON-1 was back to age-matched (8-week-old) Wistar values after IL-1Ra *in vivo* (data not shown). As expected, IL-1Ra treatment reduced islet hyper-vascularisation and islet fibrosis in GK rats. These data highlight the primary role of IL-1 in the pathogenesis of islet microangiopathy in a spontaneous T2D model (Fig. 3). In this regard, co-expression of IL-1Ra and VEGF improves human islet survival, which is strictly dependent upon adequate revascularization [119].

Concerning IL-1Ra-induced islet endocrine modifications, we recently described elsewhere [52]: 1) no change of the percentage of pancreatic β-cell area between sham (saline) and IL-1ra-treated GK rats, which would be in agreement with data published on the high fat diet (HFD) fed mouse [30]; 2) IL-1Ra enhancement of the expression of insulin processing enzymes, proconvertase 1 and 2, concomitant with increased insulin gene expression (INS1 and INS2): indeed, IL-1β downregulates both proconvertase expression, impairing insulin processing, either alone or in combination with other cytokines such as IL-6 and TNF-α [120]–[123]. In GK rats, IL-1Ra reduces islet IL-1β, IL-6, and TNF-α mRNAs and IL-6 production, likely improving insulin processing, as reflected by their better circulating pro-insulin/insulin ratio.

The beneficial effects of IL-1Ra concerning GK glucose homeostasis appears to be somehow limited based on the glycemia values measured at the end of treatment, as shown here. However: 1) the effect of IL-1Ra on glycemia varied greatly from one day to another, despite our attempt to control as best as possible environmental conditions, but the highest dose of IL-1Ra (100 mg/kg/day) administered during 1 month decreased the AUC by 50% [52]; 2) glycemia is the result of insulin production and action and involvement of counterregulatory hormones, notably from from α-cells. In this regard, because increased IL-6 release by GK islets may have increase their α-cell mass, as described in response to HFD in mice [124], IL-1Ra, which reduced GK islet IL-6 release, might have consequently diminish their α-cell mass. However, adult Wistar and GK rats have similar α-cell mass and glucagonemia [125] and 100 mg/kg/day IL-1Ra *in vivo* did not modify GK glucagonemia (data not shown); 3) IL-1Ra appears less effective on insulin resistance at higher dose [52]; 4) the time of treatment onset, the dose and the duration of treatment could be improved; 5) TLR activation by FFA may be concomitantly at work in GK rats and IL-1Ra treatment did not modify circulating GK lipid parameters [52].

Last but not least, the drastic under-expression of the soluble epoxide hydrolase (sEH) might represent the major attempt to compensate the defects triggered by metabolically-induced inflammation in GK islets. Indeed, sEH is implicated in the metabolism of epoxyeicosatrienoic acids (EETs) (see for reviews, [126]–[128]). These EETs are derived from arachidonic acid by cytochrome P450 epoxygenases. Their degradation by sEH generate dihydroxyeicosatrienoic acids, which are less active than their parent epoxides. Decreased sEH activity would therefore be expected to increase intracellular EET levels and prolong their beneficial effects, which include: 1) potent vasodilatation; 2) marked anti-inflammatory action [129]; 3) antioxidant effect by inducing the expression of a set of antioxidant genes, including thioredoxin and superoxide dismutase, as described in GK rat islets [44]; 4) beneficial effects on vessels: EETs inhibit the migration and proliferation of VSMC and, by contrast, stimulate EC proliferation and angiogenesis, particularly in response to hypoxia [127]. Very recent data showed that sEH inhibitors attenuate the progression of renal damage in diabetic GK rats from Taconic and also the development of atherosclerosis in apolipoprotein-E-knockout mice [130], [131]. Moreover, it has been described several polymorphisms in the gene encoding human sEH that encode variants with altered catalytic activity. Some of these variants are associated with increased risk of atherosclerosis (for review, see [127]).

In conclusion, these data offer a better understanding of the pathophysiology of islet behaviour in a spontaneous T2D animal model, where both pro- and anti-vasoconstrictor, pro- and antioxidant, pro- and anti-inflammatory, and pro- and anti-angiogenic mechanisms are concomitantly at play in islets during disease progression. The protective mechanisms may thus explain long-lasting mild hyperglycemia in GK rats, despite early islet endothelial activation associated with inflammation and OS. These data also highlight the crucial role of IL-1 in triggering islet OS. Therefore, counteracting endothelial cell inflammation as early as possible is one way to prevent OS-related disorders in type 2 diabetes pathophysiology.

## Methods

### Animals

All animal experiments were conducted on fed age-matched male GK and nondiabetic Wistar rats from our local colonies (Paris, France) in accordance with accepted standards of animal care, established by the French National Center for Scientific Research. Characteristics of the nonobese GK rat model of T2D maintained in our colony at the University Paris-Diderot together with the Wistar control rats have been previously described [32]. Two and a half-month-old male rats were killed by decapitation and blood and pancreata collected for measurement of metabolic parameters, islet isolation and quantitative RT-PCR analysis or pancreas immunohistochemistry.

### Metabolic parameters

Basal morning glycemia was determined with a glucometer. Serum insulin was assayed by ELISA (Mercodia). FFA levels were quantified using an enzymatic colorimetric assay (NEFA C) (Wako Chemicals GmbH). Cholesterol, HDL, and triglyceride serum levels were determined by using colorimetric assays (Penta Cholesterol CP kit, HDL cholesterol direct kit and Pentra Triglycerid CP kit, respectively, ABX Diagnostics). To measure glutathione, 50 µl of RBC were added to 450 µl of a mixture (1∶5 v/v) of EDTA (1%)/metaphosphoric acid (5%), and 100 or 200 islets were mixed with 5% metaphosphoric acid (300 µl). After centrifugation (3000 g, 10 min, 4°C), reduced glutathione (GSH) and its oxidized form (GSSG) were identified in supernatants by reverse-phase HPLC with electrochemical detection [132]). Total glutathione content, referred to as “equivalent GSH” (Eq GSH), is the sum of GSH and doubled GSSG concentrations (2GSH→GSSG). The glutathione redox state is: [total forms] ×100, with [total forms]  =  [oxidized form]+[reduced form]. Alpha-tocopherol was determined in heparinated plasma (100 µl) extracted with 2-propanol (400 µl) [133]. Plasma homocysteine was assayed by using the fluorimetric HPLC method previously described [134]. PON-1 activity assay was performed on 5 µl of plasma. PON-1 arylesterase activity toward phenyl acetate was quantified spectrophotometrically using 20 mM of Tris-HCl, pH 8.3 with 1 mM of CaCl_2_ and 10 mM of phenyl acetate (Sigma-Aldrich). The reaction was performed at room temperature for 1 min by measuring the appearance of phenol at 270 nm with the use of continuously and automated recording spectrophotometer. All values were corrected for non-enzymatic hydrolysis. Serum leptin, cytokines and chemokines were assayed using Luminex™ (Millipore, Switzerland) [124].

### Islet isolation and mRNA analysis

Pancreatic islets were isolated using collagenase (Boehringer Mannheim) and then handpicked under a stereomicroscope [135]. Total RNA was isolated from islets using the RNeasy mini kit (Qiagen) and its concentration determined by optical density at 260 nm. To remove residual DNA contamination, the RNA samples were treated with RNAse-free DNAse (Qiagen) and purified with RNeasy mini-column (Qiagen). Total RNA (4 µg) from each islet sample was reverse transcribed with 40 U of M-MLV Reverse Transcriptase (Invitrogen) using random hexamer primers. The primers used were derived from rat sequences and designed using OLIGO6 (see Table 3). Quantitative RT-PCR amplification reactions were carried out in a LightCycler 1.5 detection system (Roche) using the LightCycler FastStart DNA Master plus SYBR Green I kit (Roche). Reverse transcribed RNA (10 ng) was used as the template for each reaction. All reactions were run in duplicate with no template control. The PCR conditions were: 95°C for 10 min, followed by 40 cycles at 95°C for 10 s, 60°C for 10 s and 72°C for 10 s. Changes in mRNA expression were calculated using difference of C_T_ values as compared to a housekeeping gene (rpL19 or Ef1a), and expressed relative to controls.

**Table 3:** Primer oligonucleotide sequences of selected genes.

Gene		Sequences
Ace1	Forward:	GCGGAGTCGATGCTGGAGAA
Ace1	Reverse:	GTGGCCCATCTCGTGGTGTA
Agt	Forward:	CTCCCAGAGCCAACCTTTGA
Agt	Reverse:	CAGCATCTTGTACATGCGGAAA
Agtr1a	Forward:	CTGGCAGAAATGCAATCTCATCA
Agtr1a	Reverse:	GCCCTTTGGGAGTTGAACAGAA
Alox15	Forward:	GGGCCACTGCTGTTCGTAAGA
Alox15	Reverse:	GCCCTGAACCCATCGGTAA
Casp 1	Forward:	CCTGTGCGATCATGTCACTAA AA
Casp 1	Reverse:	GCCAGGTAGCAGTCTTCATTACAA
Cat	Forward:	GGGTGGTGCTCCCAACTACTA
Cat	Reverse:	CACCTGAGTGACGTTGTCTTCA
Cd53	Forward:	GCGTGGTTTCACTCCAATTTC
Cd53	Reverse:	GGACATCCCCAGCACCTGTA
Cd74	Forward:	CCAGGACCACGTGATGCA
Cd74	Reverse:	CCCCTTCAGCTGTGGGTAGTT
Col1a1	Forward:	CCCAACCCCCAAAAA
Col1a1	Reverse:	CTGCGTCTGGTGATACATATTCTTCT
Col3a1	Forward:	AGCTGGCCTTCCTCAGACTTC
Col3a1	Reverse:	GCTGTTTTTGCAGTGGTATGTAAT
Cox2	Forward:	CGCTTCTCCCTGAAACCTTACA
Cox2	Reverse:	GGAGAATGGAGCTCCAAGTTCTA
Edn1	Forward:	GCCAGTGTGCTCACCAAAAAGA
Edn1	Reverse:	GGACAGGGTTTTCCCTTCTTGAA
eNos	Forward:	CACCCGGACAACCTCATCA
eNos	Reverse:	CTGCTCATTTTCCAAGTGCTTCA
Ephx2	Forward:	ACCCATCGGTGACCTCCAA
Ephx2	Reverse:	AAGGCCACGTCAGAAATGAAA
Fn1	Forward:	CCTACGGATGACTCATGCTTT
Fn1	Reverse:	CAGATAACCGCTCCCATTCC
Gclc	Forward:	GCCGTGGTGGATGGGTGTA
Gclc	Reverse:	CCACGTCGACTTCCATGTTTTCA
Gpx1	Forward:	GTGCGAGGTGAATGGTGAGAA
Gpx1	Reverse:	CTGGACCTACCAGGAACTTCTCAAA
GRO1/KC	Forward:	GGAAGAAGGGCGGAGAGATGA
GRO1/KC	Reverse:	CCTCTCACACATTCCTCACCCTAA
Gsr	Forward:	GCCCACAGCGGAAGTCAA
Gsr	Reverse:	GGGCAAGTCTTCCAGCTGAAA
H2-Ea	Forward:	CCCTCC AGCGGTCAATGT
H2-Ea	Reverse:	TGACACGCCTTTGGTGACA
Hmox1	Forward:	GAGACGCCCCGAGGAAA
Hmox1	Reverse:	GGGCCAACACTGCATTTACA
Hif1α	Forward:	GGCGACATGGTTTACATTTCTGATAT
Hif1α	Reverse:	GCTCCGCTGTGTGTTTAGTTCTTT
iNos	Forward:	CGCTACACTTCCAACGCAACA
iNos	Reverse:	CGGATTCTGGAGGGATTTCA
Icam1/CD54	Forward:	CGGGAGATGAATGGTACCTACAA
Icam1/CD54	Reverse:	CCGCAATGATCAGTACCAACA
Il1ra	Forward:	GAGGAACAATTTTTGCAGGGTGTA
Il1ra	Reverse:	CCCAGAGGGCAGAGGCAATA
Il1β	Forward:	CTGGTACATCAGCACCTCTCAA
Il1β	Reverse:	GAGACTGCCCATTCTCGACAA
Il6	Forward:	GCCACTGCCTTCCCTACTTCA
Il6	Reverse:	GACAGTGCATCATCGCTGTTCA
Mip1α/Ccl3	Forward:	CCAAGTCTTCTCAGCGCCATA
Mip1α/Ccl3	Reverse:	GCAGATCTGCCGGTTTCTCTTA
Mcp1/Ccl2	Forward:	CTGGACCAGAACCAAGTGAGATCA
Mcp1/Ccl2	Reverse:	GTGCTTGAGGTGGTTGTGGAAA
Myd88	Forward:	CGGAGGAGATGGGTTTCGAGTA
Myd88	Reverse:	CGATGCGGTCCTTCAGTTCATA
Nox2	Forward:	CTGGACATCCTGGTGGTTTTCA
Nox2	Reverse:	GGACCGCATCATGGTGAAGAA
NfκB/Rela	Forward:	CTGGCCATGGACGATCTGTTT
NfκB/Rela	Reverse:	CCCTCGCACTTGTAACGGAAA
Nrf2	Forward	CCACGTTGAGAGCTCAGTCTTCA
Nrf2	Reverse	GACACTGTAACTCGGGAATGGAAA
Pai1/Serpine1	Forward:	CCGACCAAGAGCAGCTCTCTGTA
Pai1/Serpine1	Reverse:	GTGCCGAACCACAAAGAGAAA
Pecam1/Cd31	Forward:	GGCCCTGTCACGTTTCAGTTTTA
Pecam1/Cd31	Reverse:	CCTGCTCCTTGCTAGTTTGTTCA
Prdx1	Forward:	GCATGGATTAACACACCCAAGA
Prdx1	Reverse:	GCCCCTGAAAGAGATACCTTCA
Ptgis	Forward:	CGCTGGCTACCTGACCCTGTA
Ptgis	Reverse:	GCCAGTTTGGGGAGCATCA
Sele/Cd62	Forward:	GCCAGCCCTCTACCAGAATGA
Sele/Cd62	Reverse:	CCCAAATTCCAGAGTGACGAAGA
Sod1	Forward:	GCCGTGTGCGTGCTGAA
Sod1	Reverse:	GCCTTGTGTATTGTCCCCATA
Sod2	Forward:	GGCCAAGGGAGATGTTACAA
Sod2	Reverse:	GACCCAAAGTCACGCTTGA
Tgfβ1	Forward:	GAGCCCGAGGCGGACTACTA
Tgfβ1	Reverse:	CCCGAATGTCTGACGTATTGAAGA
Tlr2	Forward:	Rn02133647_s1 These were purchased from AB, this is the assay number, the rest is proprietary.
Tlr2	Reverse:	Rn02133647_s1
Tlr4	Forward:	CGCTTTCAGCTTTGCCTTCA
Tlr4	Reverse:	GCCAGAGCGGCTACTCAGAAA
Tnfα	Forward:	GGGGCCTCCAGAACTCCA
Tnfα	Reverse:	GGAGCCCATTTGGGAACTTCT
Txd1	Forward:	GGATGTTGCTGCAGACTGTGAA
Txd1	Reverse:	GGCTTCGAGCTTTTCCTTGTTA
Vcam1/CD106	Forward:	GCTCTTGTTTGCCTCGCTAA
Vcam1/CD106	Reverse:	GTGGGTTCTTTCGGAGCAA
Vegfa	Forward:	CCAGGAGTACCCCGATGAGATAGA
Vegfa	Reverse:	GGTGAGGTTTGATCCGCATGA

### In vivo IL-1Ra treatment

IL-1Ra (kindly donated by Amgen, CA, USA) treatment of GK rats was performed by twice daily subcutaneous injections (100 mg/kg/day). Treatment was initiated 3 days following weaning at 4 weeks of age, i.e., after onset of mild fed hyperglycemia [32]. IL-1Ra treatment, which was given during growth of the animals, had no effect on body weight. Subcutaneous (s.c.) injection experiments were stopped 4 weeks after initiation of treatment and the animals sacrificed for islet isolation followed by quantitative RT-PCR or pancreas immunohistochemistry.

### Immunohistochemistry

GK rat pancreatic cryosections were incubated with rabbit anti-fibronectin (1/40, Novotec) or rabbit anti-VWF (1/100, Dako), followed by swin anti-rabbit secondary antibody (1/100, Dako) as previously described [6]. Antibody-stained surface areas were quantified blindly by measuring the surface area labeled by each marker in a given islet and expressing it to the whole surface of this islet (in 25–40 islets in 3 animals per treatment group), using an Olympus BX40 microscope.

### Statistics

Data are presented as means±SEM. Statistical analyses used an unpaired Student's *t*-test or ANOVA as appropriate. Significance was defined as p<0.05.
